# Outcomes After Decompressive Surgery for Severe Cerebral Venous Sinus Thrombosis Associated or Not Associated with Vaccine-Induced Immune Thrombosis with Thrombocytopenia: A Multicenter Cohort Study

**DOI:** 10.1007/s12028-023-01782-6

**Published:** 2023-07-27

**Authors:** Johann Otto Pelz, Martin Kenda, Angelika Alonso, Nima Etminan, Matthias Wittstock, Wolf-Dirk Niesen, Johann Lambeck, Erdem Güresir, Johannes Wach, Tim Lampmann, Rainer Dziewas, Markus Wiedmann, Hauke Schneider, Antonios Bayas, Monika Christ, Annerose Mengel, Sven Poli, Dirk Brämer, Dirk Lindner, Christian Pfrepper, Christian Roth, Farid Salih, Albrecht Günther, Dominik Michalski, for the IGNITE Study Group

**Affiliations:** 1 Department of Neurology, University Hospital Leipzig, Liebigstrasse 20, 04103 Leipzig, Germany; 2 Department of Neurology and Experimental Neurology, Charité-Universitätsmedizin Berlin, Campus, Virchow-Klinikum, Berlin, Germany; 3 Department of Neurology, Medical Faculty Mannheim, University of Heidelberg, Mannheim, Germany; 4 Department of Neurosurgery, Medical Faculty Mannheim, University of Heidelberg, Mannheim, Germany; 5 Department of Neurology, University Medicine Rostock, Rostock, Germany; 6 Department of Neurology and Clinical Neurophysiology, University Medical Center Freiburg, University of Freiburg, Freiburg, Germany; 7 Department of Neurosurgery, University Hospital Bonn, Bonn, Germany; 8 Department of Neurosurgery, University Hospital Leipzig, Leipzig, Germany; 9 Department of Neurology and Neurorehabilitation, Klinikum Osnabrueck, Osnabrueck, Germany; 10 Department of Neurosurgery, Oslo University Hospital, Oslo, Norway; 11 Department of Neurology, University Hospital Augsburg, Augsburg, Germany; 12 Department of Neurology and Stroke, University Hospital Tuebingen, Eberhard-Karls University, Tuebingen, Germany; 13 Department of Neurology, Jena University Hospital, Jena, Germany; 14 Division of Haemostaseology, Medical Department I, University Hospital Leipzig, Leipzig, Germany; 15 Department of Neurology, Klinikum Kassel, Kassel, Germany; 16 Medizinische Fakultät Carl Gustav Carus, Technische Universität Dresden, Dresden, Germany

**Keywords:** Cerebral venous sinus thrombosis, Decompressive surgery, Vaccine-induced immune thrombosis with thrombocytopenia, VITT, COVID-19, Vaccination, Space-occupying infarction

## Abstract

**Background:**

Clinical observations indicated that vaccine-induced immune thrombosis with thrombocytopenia (VITT)-associated cerebral venous sinus thrombosis (CVST) often has a space-occupying effect and thus necessitates decompressive surgery (DS). While comparing with non-VITT CVST, this study explored whether VITT-associated CVST exhibits a more fulminant clinical course, different perioperative and intensive care unit management, and worse long-term outcome.

**Methods:**

This multicenter, retrospective cohort study collected patient data from 12 tertiary centers to address priorly formulated hypotheses concerning the clinical course, the perioperative management with related complications, extracerebral complications, and the functional outcome (modified Rankin Scale) in patients with VITT-associated and non-VITT CVST, both with DS.

**Results:**

Both groups, each with 16 patients, were balanced regarding demographics, kind of clinical symptoms, and radiological findings at hospital admission. Severity of neurological symptoms, assessed with the National Institute of Health Stroke Scale, was similar between groups at admission and before surgery, whereas more patients with VITT-associated CVST showed a relevant midline shift (≥ 4 mm) before surgery (100% vs. 68.8%, *p* = 0.043). Patients with VITT-associated CVST tended to undergo DS early, i.e., ≤ 24 h after hospital admission (*p* = 0.077). Patients with VITT-associated CVST more frequently received platelet transfusion, tranexamic acid, and fibrinogen perioperatively. The postoperative management was comparable, and complications were evenly distributed. More patients with VITT-associated CVST achieved a favorable outcome (modified Rankin Scale ≤ 3) at 3 months (*p* = 0.043).

**Conclusions:**

Although the prediction of individual courses remains challenging, DS should be considered early in VITT-associated CVST because an overall favorable outcome appears achievable in these patients.

## Background

Cerebral venous sinus thrombosis (CVST) is a rare disease with an overall yearly incidence of 1.32 to 1.9 per 100,000 people [1, 2]. As potential complications, the thrombotic occlusion of cerebral sinus or veins may result in cerebral edema, intracerebral congestive hemorrhage, or ischemic stroke.

To prevent the progress of thrombosis, standard therapy involves early anticoagulation with low molecular weight heparins or unfractionated heparin, followed by oral anticoagulation for a certain period [3]. Despite early anticoagulation, up to 8.3% of patients with CVST may die, mostly because of a critical increase in the intracranial pressure (ICP) due to progressive intracerebral bleeding or cerebral edema [4, 5]. Decompressive surgery (DS) accompanied by sufficient medical ICP lowering therapy (i.e., deep analgosedation, osmotherapy) is considered as an ultima ratio approach to save a patient’s life [6]. However, in contrast to space-occupying cerebral edema caused by ischemic stroke [7], evidence for this approach in patients with CVST is limited to retrospective case series [8]. In the largest cohort summarizing data from a single center, Zhang and colleagues [9] reported a mortality of 14% in 58 patients with space-occupying CVST treated with DS.

In 2021, an increased number of CVSTs was noted shortly after people had been vaccinated with vector vaccines (in particular with ChAdOx1 nCOV-19 from AstraZeneca or with AD26.COV2S from Johnson & Johnson) against the severe adult respiratory syndrome–coronavirus 2 (SARS-CoV 2) [10, 11]. These cases were characterized by a strong activation of the coagulation system with low platelets, low fibrinogen, and a high D-dimer [12]. Covering this syndrome, the term “vaccine-induced immune thrombosis with thrombocytopenia” (VITT) was introduced. Initially, mortality of patients with VITT-associated CVST was reported to be dramatically high, ranging from 29 to 61% [13, 14]. Meanwhile, the immune process underlying the cause of VITT was elucidated in detail, allowing a more specific treatment regime with anticoagulation and immunomodulation [15–17]. Specific treatment as well as early identification may have contributed to a declining mortality of patients with VITT [18–20]. However, despite these advances in treating patients with VITT-associated severe CVST, case series continued reporting high rates of fatal courses in patients with VITT-associated CVST and DS, which has led to the impression that this approach could not influence the fatal course [21, 22].

So far, there is no systematic investigation on the course of VITT-associated CVST with space-occupying effects and the need for DS compared with severe CVST without evidence for VITT. Using a multicenter approach, this study aimed to explore the clinical course, radiological and periprocedural parameters, and the functional outcome of patients suffering from VITT-associated CVST with space-occupying effects and the need for surgical treatment compared to those with non-VITT CVST and DS, too.

## Methods

### Study Design and Content

This retrospective, noninterventional, multicenter cohort study was performed according to the ethical standards laid down in the 1964 Declaration of Helsinki and its later amendment. The study was approved by the local ethics committee of the University Hospital Leipzig, Leipzig, Germany (reference number 409/21-ek). If applicable, participating centers obtained the individual approval of the local ethics committee.

Because of the overall low number of patients suffering from VITT-associated CVST with consecutive DS, this study was initiated within the Initiative of German Neurointensive Trial Engagement (IGNITE) group. Based on clinical experiences of the IGNITE group while treating patients with non-VITT-associated CVST, hypotheses were formulated a priori*,* and an electronic case report form was used to collect data addressing these hypotheses in patients with VITT-associated and non-VITT CVST, both with a need for DS.

### Main Hypotheses

This study aimed to address the following hypotheses: (1) Patients with VITT-associated CVST exhibit a more fulminant clinical course than patients with non-VITT CVST. (2) Because of the massive activation of the coagulation system in patients with VITT-associated CVST, their perioperative management differs from those with non-VITT CVST. (3) Extracerebral complications during the treatment in the neurointensive care unit (neuro-ICU) or intensive care unit (ICU) are more frequent in patients with VITT-associated CVST. (4) The functional outcome, indicated by the proportion of patients achieving a modified Rankin Scale (mRS) of ≤ 3 after 3 months, does not differ significantly.

### Inclusion Criteria and Data Collection

Patients were eligible for this study if they had radiological evidence for CVST (magnetic resonance angiography, computed tomography angiography, or digital subtraction angiography) and had undergone DS because of a space-occupying effect of the hemorrhage or the edema formation related to CVST. Patients were included if treated between January 1st, 2012, and July 31st, 2021, while the period prior to 2021 was necessary to recruit enough patients for the non-VITT CVST group.

In addition, patients assigned to VITT-associated CVST had to fulfill the diagnostic criteria for a definite or probable VITT according to the interim case definition of the Brighton Collaboration and the criteria that were applied by Perry and colleagues [13, 23]: (1) Vaccination with either ChAdOx1 nCOV-19 from AstraZeneca or AD26.COV2S from Johnson & Johnson 4 to 30 days before the diagnosis of CVST. (2) Typical laboratory findings including low platelets (< 150 × 10^9^/l) and a markedly elevated D-dimer. (3) Detection of anti-PF4 antibodies by enzyme-linked immunoassay (ELISA) or functional assay.

The electronic case report form included demographic data, data about the functional status before vaccination, at hospital admission, at discharge, and, if available, after 3 months; laboratory parameters with a particular focus on the coagulation system at hospital admission and before surgery; clinical data at hospital admission, before surgery, and during ICU stay; neuroradiological data at hospital admission and before surgery; and perioperative details, overall extracted from medical records.

### Statistical Analysis

The software SPSS (version 27.0; IBM Corp, Armonk, NY) was used for statistical calculations. After descriptive analyses, statistical significance between groups was assessed by Fisher’s exact test for categorial variables and, because of the small sample size, by the Mann–Whitney *U*-test for interval-scaled parameters. Thereby, a *p* value ≤ 0.05 was defined as statistically significant. In case of multiple comparisons between groups, the Bonferroni-Holm correction was used to correct for multiple *p* value testing.

## Results

Twelve tertiary hospitals in Germany and Norway participated in this cohort study and provided data from 16 patients with VITT-associated CVST (inclusion period January 1st, 2021, to July 31st, 2021; 45.8 ± 11.8 years, 9 [56.3%] women) and 16 patients with non-VITT CVST (inclusion period January 1st, 2012, to July 31st, 2021; 43.4 ± 15.1 years, 9 [56.3%] women), both requiring DS.

Comparing 16 patients with VITT-associated CVST and 16 patients with non-VITT CVST included in the study, baseline demographic data and typical risk factors for CVST were well balanced between both groups (Table 1). Both groups had a mean pre-mRS of 0 and 0.2 (range 0 to 2). Patients with VITT-associated CVST received SARS-CoV 2 vaccination on average 11 (range 5 to 26) days before hospital admission. They reported the first headache on average 8.6 days (range 4 to 18) after vaccination, resulting in a time interval of about 3 days between the first headache and hospital admission (Table 1). Regarding the clinical and functional state at hospital admission, patients with VITT-associated and non-VITT CVST were equally impaired, as indicated by a similar National Institute of Health Stroke Scale score (NIHSS), level of consciousness, Simplified Acute Physiology Score (SAPS) 2, and Acute Physiology and Chronic Health Evaluation (APACHE) 2 score. Between hospital admission and DS, patients of both groups showed neurological deterioration as demonstrated by a significant increase of the NIHSS (patients with VITT-associated CVST + 8.5, *p* = 0.012; patients with non-VITT CVST + 5.5, *p* = 0.028), and a decreasing level of consciousness (Table 2). However, most radiological findings concerning cerebral edema and hemorrhage were comparable between both groups at hospital admission and before surgery, while proportions of subarachnoid hemorrhage and relevant midline shift (≥ 4 mm) were slightly increased in patients with VITT-associated CVST (Tables 2 and 3).

**Table 1:** Baseline demographic data, overview of risk factors for CVST, and timeline of the prehospital course in patients with VITT-associated CVST and non-VITT CVST

Demographic	VITT-associated CVST (*n* = 16)	Non-VITT CVST (*n* = 16)	*p* value
Age, M ± SD (years)	45.8 ± 11.8	43.4 ± 15.1	0.491
Gender (women), *n* (%)	9 (56.3)	9 (56.3)	1.0
pre-mRS (M ± SD)	0	0.2 ± 0.5	0.564
Prior COVID-19, *n* (%)	No: 13 (81.3) unknown: 3 (20)	No: 16 (100)	–
Intake of drugs affecting coagulation system in before, *n* (%)	2 (12.5)	1 (6.3)	1.000
Arterial hypertension, *n* (%)	4 (25.0)	1 (6.3)	0.333
Smoking, *n* (%)	2 (12.5)	3 (18.8)	1.000
Thrombophilic diathesis, *n* (%)	0	3 (18.8)	0.226
Hormonal contraception/replacement, *n* (%)	5 (55.6% of women)	3 (33.3% of women)	0.685
Anemia, *n* (%)	1 (6.3)	2 (12.5)	1.000
Initial hospital admission to tertiary hospital, *n* (%)	10 (62.5)	9 (56.3)	1.000
Time from vaccination to beginning of headache, M ± SD (*n*) (days)	8.6 ± 3.8 (13)	–	–
Time from vaccination to first neurological deficits, M ± SD (*n*) (days)	11.1 ± 5.2 (16)	–	–
Time from vaccination to hospital admission, M ± SD (*n*) (days)	11.1 ± 5.1 (16)	–	–
Time from the beginning of headache to hospital admission, M ± SD (*n*) (days)	3.0 ± 3.0 (13)	2.5 ± 1.8 (8)	1.0

*COVID-19* coronavirus disease of 2019, *CVST* cerebral venous sinus thrombosis, *M* mean, *mRS* modified Rankin Scale, *SD* standard deviation, *VITT* vaccine-induced immune thrombosis with thrombocytopenia

**Table 2:** Overview of the clinical affection, radiological findings, and parameters of the coagulation system in patients with VITT-associated CVST and non-VITT at hospital admission and before surgery

Parameter	VITT-associated CVST (*n* = 16)	Non-VITT CVST (*n* = 16)	*p* value
mRS at hospital admission, M ± SD (points)	3.1 ± 2.0	4.3 ± 1.1	0.072
NIHSS at hospital admission, M ± SD (points)	12.6 ± 14.2	14.8 ± 9.8	0.310
NIHSS before surgery, M ± SD (points)	22.4 ± 11.6	21.3 ± 7.9	0.974
Change of NIHSS between hospital admission and surgery, M ± SD (points)	8.5 ± 8.7	5.5 ± 6.3	0.456
Level of consciousness at hospital admission, *n* (%)	Awake: 9 (56.3)	Awake: 7 (43.8)	–
	Somnolence: 1 (6.3)	Somnolence: 1 (6.3)	
	Sopor: 1 (6.3)	Sopor: 2 (12.5)	
	Coma: 5 (31.3)	Coma: 6 (37.5)	
Level of consciousness before surgery, *n* (%)	Awake: 1 (6.3)	Awake: 1 (6.3)	–
	Somnolence: 1 (6.3)	Somnolence: 1 (6.3)	
	Sopor: 5 (31.3)	Sopor: 4 (25.0)	
	Coma: 9 (56.3)	Coma: 10 (62.5)	
APACHE 2 score at hospital admission, M ± SD (points)	18.4 ± 9.9	13.1 ± 8.6	0.238
SAPS 2 at hospital admission, M ± SD (points)	44.0 ± 20.2	31.7 ± 20.6	0.123
Patients with midline shift (≥ 4 mm) before surgery, *n* (%)	16/16 (100)	11/16 (68.8)	0.043
Maximum midline shift before surgery, M ± SD (mm)	10.6 ± 3.2	7.8 ± 6.8	0.294
Dilated pupils or anisocoria before surgery, *n* (%)	8/16 (50.0)	8/16 (50)	1.0
Dilated pupils or anisocoria after surgery, *n* (%)	8/16 (50.0)	3/16 (18.8)	0.135
Proportion of patients with a period of less than 24 h between hospital admission and DS, *n* (%)	10/16 (62.5)	5/16 (31.3)	0.078
Platelet count at hospital admission, M ± SD (× 10^9^/l)	54 ± 46	230 ± 57	< 0.001
Platelet count before surgery, M ± SD (× 10^9^/l)	57 ± 25	253 ± 160	< 0.001
D-dimer at hospital admission, M ± SD (mg/l)	30.0 ± 10.7	2.9 ± 1.7	< 0.001
D-dimer before surgery, M ± SD (mg/l)	29.9 ± 9.9	1.7 ± 1.5	< 0.001
Fibrinogen at hospital admission, M ± SD (g/l)	2.7 ± 2.7	3.1 ± 0.8	0.237
Fibrinogen before surgery, M ± SD (g/l)	2.2 ± 1.3	3.5 ± 0.8	0.041
Activated partial thromboplastin time at hospital admission, M ± SD (s)	28.0 ± 5.0	27.9 ± 11.1	0.379
Activated partial thromboplastin time before surgery, M ± SD (s)	36.8 ± 20.3	41.4 ± 21.5	0.697
Prothrombin time at hospital admission, M ± SD (%)	80.0 ± 26.7	91.9 ± 17.6	0.201
Prothrombin time before surgery, M ± SD (%)	65.5 ± 20.0	83.6 ± 14.7	0.020

*APACHE* Acute Physiology and Chronic Health Evaluation, *CVST* cerebral venous sinus thrombosis, *M* mean, *mRS* modified Rankin Scale, *NIHSS* National Institute of Health Stroke Scale, *SAPS* Simplified Acute Physiology Score, *SD* standard deviation, *VITT* vaccine-induced immune thrombosis with thrombocytopenia

**Table 3:** Radiological findings in patients with VITT-associated CVST in comparison with patients without VITT

Finding	VITT-associated CVST (*n* = 16)	Non-VITT CVST (*n* = 16)	*p* value
Cerebral edema on first cerebral imaging, *n* (%)	8/16 (50.0)	12/16 (75)	0.273
Cerebral congestive / parenchymal hemorrhage on first cerebral imaging, *n* (%)	12/16 (75.0)	12/16 (75)	1.0
Subarachnoid hemorrhage on first cerebral imaging, *n* (%)	3/16 (18.8)	8/16 (50)	0.135
Sub-/epidural hemorrhage on first cerebral imaging, *n* (%)	3/16 (18.8)	3/16 (18.8)	1.0
Any intracranial hemorrhagic lesion on first cerebral imaging, *n* (%)	12/16 (75.0)	14/16 (87.5)	0.651
Progression of edema on last cerebral imaging before surgery, *n* (%)	10/16 (62.5)	10/16 (62.5)	1.0
Progression of congestive/parenchymal hemorrhage on last cerebral imaging before surgery, *n* (%)	11/16 (68.8)	6/15 (37.5)	0.213
Progression of existing or new subarachnoid hemorrhage on last cerebral imaging before surgery, *n* (%)	7/14 (50.0)	0/16	< 0.001
Progression of existing or new sub-/epidural hemorrhage on last cerebral imaging before surgery, *n* (%)	2/14 (14.3)	1/16 (6.3)	0.903
Any intracranial hemorrhagic lesion on last cerebral imaging before surgery, *n* (%)	13/14 (92.9)	15/16 (93.8)	1.0

*CVST* cerebral venous sinus thrombosis, *VITT* vaccine-induced immune thrombosis with thrombocytopenia

The acute treatment after hospital admission comprised, for instance, the initiation of anticoagulation and, in a few cases, venous mechanical thrombectomy, which was also comparable between both groups (Table 4). In the VITT-associated CVST group, treatment with intravenous immunoglobulins was started on average 13.5 h after admission, whereas this was naturally not done in the non-VITT group. Patients with VITT-associated CVST tended to undergo surgical treatment early, i.e., within 24 h after hospital admission (62.5% vs. 31.3%, *p* = 0.078, Table 2), and their anticoagulation was stopped closer to surgery than in patients with non-VITT CVST (1.9 h vs. 3.7 h, *p* = 0.055, Table 4).

**Table 4:** Comparison of acute and intraoperative/perioperative treatment of patients with VITT-associated CVST and patients with non-VITT CVST

Parameter	VITT-associated CVST (*n* = 16)	Non-VITT CVST (*n* = 16)	*p* value
Patients with therapeutic anticoagulation before surgery, *n* (%)	14/16 (87.5)	16/16 (100)	0.484
Argatroban	11/16 (68.8)	0	
Fondaparinux	1 (6.3)	0	
Low molecular weight heparins	3/16 (18.8)	2/16 (12.5)	
Unfractionated heparin	0	14/16 (87.5)	
Missing data	1/16 (6.3)	0	
Time from hospital admission to beginning of anticoagulation, M ± SD (h)	5.8 ± 5.0 (*n* = 12)	6.8 ± 6.6	0.767
Patients with immunomodulatory treatment, *n* (%)	15/16 (93.8)	–	–
Immunoglobulins	13/16 (81.3)	–	–
Corticosteroids	3/16 (18.8)	–	–
Plasma exchange	0	–	–
Other	1/16 (6.3)	–	–
Time from hospital admission to beginning of immunomodulatory treatment, M ± SD (h)	13.5 ± 19.0 (*n* = 13)	–	–
Venous mechanical thrombectomy, *n* (%)	3/16 (18.8)	5/16 (31.3)	0.685
Stopping anticoagulation before surgery, *n* (%)	7/14 (50.0)	9/13 (69.2)	0.532
Time from stopping anticoagulation to surgery, M ± SD (h)	1.9 ± 1.9 (*n* = 7)	3.7 ± 3.3 (*n* = 9)	0.055
Kind of DS, *n* (%)	Unilateral: 14 (87.5)	Unilateral: 12 (75.0)	–
	Bilateral: 0	Bilateral: 3 (18.8)	
	Suboccipital: 2 (12.5)	Suboccipital: 1 (6.3)	
Duration of surgery, M ± SD (minutes)	120 ± 77	152 ± 86	0.128
Intraoperative volume replacement, M ± SD (ml)	2440 ± 2057 (*n* = 13)	1983 ± 1223 (*n* = 11)	0.865
Intraoperative/perioperative replacements, *n* (%)			
Erythrocyte concentrate	3/15 (20.0)	5/16 (31.3)	0.685
Thrombocyte concentrate	10/15 (66.7)	3/16 (18.8)	0.003
Tranexamic acid	7/15 (43.8)	1/16 (6.3)	0.015
Desmopressin	1/14 (7.1)	1/16 (6.3)	1.0
PCC	4/16 (25.0)	3/16 (18.8)	1.0
Fibrinogen	5/16 (31.3)	0	0.043
Complications during surgery, *n* (%)	3/15 (20.0)	1/16 (6.3)	0.333

*CVST* cerebral venous sinus thrombosis, *DS* decompressive surgery, *M* mean, *SD* standard deviation, *PCC* prothrombin complex concentrate, *VITT* vaccine-induced immune thrombosis with thrombocytopenia

Compared with non-VITT CVST, patients with VITT-associated CVST had a lower platelet count, a higher D-dimer, and lower fibrinogen at admission and before surgery (Table 2). Thus, they received thrombocyte concentrates (66.7% vs. 18.8%), tranexamic acid (43.8% vs. 6.3%), and fibrinogen (31.3% vs. 0%) more frequently than patients with non-VITT CVST. The intraoperative volume substitution was similar between both groups. Because of missing data, the intraoperative blood loss could not be analyzed. The frequency of complications during surgery did not differ between both groups (Table 4).

Patients with VITT-associated CVST were treated more frequently in a dedicated neuro-ICU than patients with non-VITT CVST. Treatment strategies were very similar between both groups and comprised neuromonitoring (invasive ICP monitoring, external ventricular drainage), a deep analgosedation with most patients in both groups receiving up to three sedative drugs at the same time in the acute phase, osmotherapy, and targeted temperature management (Table 5). Both groups had a comparable rate of intracranial rebleeding after surgery leading to neurosurgical reintervention. Concerning general complications during the neuro-ICU stay, septic shock occurred in a slightly higher proportion in non-VITT CVST, whereas the occurrence of severe sepsis, ventilator-associated pneumonia, liver failure, acute kidney injury, delirium, and extracranial thrombosis did not differ between both groups (Table 5). The occurrence of epileptic seizures during the observation period was similar between groups (Table 6). Despite the intake of antiepileptic drugs at discharge, two of six surviving patients with VITT-associated CVST had recurrent seizures. At 3 months, most patients were still on antiepileptic medication (Table 6).

**Table 5:** Comparison of the treatment in the intensive care unit and outcome between patients with VITT-associated CVST and patients with non-VITT CVST

Parameter	VITT-associated CVST (*n* = 16)	Non-VITT CVST (*n* = 16)	*p* value
Treatment on dedicated neuro-ICU, *n* (%)	14/16 (87.5)	8/16 (50)	0.054
Total days on ICU, M ± SD (days)	11.9 ± 12.1	16.5 ± 12.9	0.305
Duration of analgosedation, M ± SD (days)	9.3 ± 12.1	11.4 ± 9.6	0.361
Maximum of concurrent drugs for analgosedation, number: *n* (%)	2: 4 (33.3)	2: 2 (12.5)	–
	3: 5 (41.7)	3: 6 (37.5)	
	4: 2 (16.7)	4: 7 (43.8)	
	5: 1 (8.3) (*n* = 12)	5: 1 (6.3)	
Duration of ventilation, M ± SD (days)	9.7 ± 14.6	14.5 ± 11.7	0.102
Invasive ICP monitoring, *n* (%)	16/16 (100)	14/16 (87.5)	0.484
External ventricular drainage, *n* (%)	7/16 (43.8)	7/16 (43.8)	1.0
Osmotherapy, *n* (%)	12/16 (75.0)	10/16 (62.5)	0.704
Targeted temperature management, *n* (%)	4/16 (25.0)	3/16 (18.8)	1.000
Intracranial rebleeding after surgery, *n* (%)	7/15 (46.7)	9/16 (56.3)	0.724
Further neurosurgical treatment in the course, *n* (%)	3/16 (18.8)	6/16 (37.5)	0.433
Severe sepsis, *n* (%)	3/14 (21.4)	6/16 (37.6)	0.440
Septic shock, *n* (%)	0/14	5/16 (31.3)	0.045
Ventilator-associated pneumonia, *n* (%)	6/14 (42.9)	6/16 (37.5)	1.000
Extracranial thrombosis, *n* (%)	3/13 (23.1)	3/16 (18.8)	1.000
Liver failure, *n* (%)	2/13 (15.4)	3/16 (18.8)	1.000
Acute kidney injury, *n* (%)	3/12 (25.0)	5/16 (31.2)	0.717
Delirium, *n* (%)	2/10 (20.0)	1/16 (6.3)	0.538
In-hospital mortality, *n* (%)	10/16 (62.5)	10/16 (62.5)	1.0
Discontinuation of treatment, *n* (%)	8/13 (61.5)	9/16 (56.25)	1.0
Proportion of good functional outcome (mRS 0–3) at 3 months, *n* (%)	5/16 (31.3)	0/16	0.043

*CVST* cerebral venous sinus thrombosis, *ICP* intracranial pressure, *ICU* intensive care unit, *M* mean, *mRS* modified Rankin Scale, *SD* standard deviation, *VITT* vaccine-induced immune thrombosis with thrombocytopenia

**Table 6:** Epileptic seizures in patients with VITT-associated CVST and patients with non-VITT CVST

Parameter	VITT-associated CVST (*n* = 16)	Non-VITT CVST (*n* = 16)	*p* value
Epileptic seizures prior to admission, *n* (%)	1/14 (7.1)	6/16 (37.5)	0.086
Epileptic seizures before surgery, *n* (%)	4/16 (25.0)	5/16 (31.3)	1.0
Epileptic seizures after decompressive surgery, *n* (%)	4/16 (25.0)	5/16 (31.3)	1.0
Intake of antiepileptic drugs at discharge, *n* (%)	4/6 (66.7)	5/6 (83.3)	1.0
New epileptic seizures since discharge, *n* (%)	2/6 (33.3)	0/5	0.455
Intake of antiepileptic drugs at 3 months, *n* (%)	4/6 (66.7)	5/6 (83.3)	1.0

*CVST* cerebral venous sinus thrombosis, *VITT* vaccine-induced immune thrombosis with thrombocytopenia

Twelve patients (six in each group) with VITT-associated or non-VITT CVST (37.5%) survived and were discharged to neurological rehabilitation. In both groups, ten patients died, most of them because of the discontinuation of treatment because of an inauspicious prognosis (Table 5). A good functional outcome after 3 months, defined as a mRS from 0 to 3, was achieved by five patients after VITT-associated CVST and no patients after non-VITT CVST and was thus significantly more frequently in patients with VITT-associated CVST (*p* = 0.043; Table 5). Mortality was equally distributed between patients with (5/8 [62.5%]) and without (15/24 [62.5%]) venous mechanical thrombectomy. All surviving patients were anticoagulated at discharge and at 3 months. Eight of the 12 surviving patients with CVST had additional venography before day 90. In two patients (25%), the initially occluded sinus or cerebral veins were reopened, and in six patients (75%), they were at least partially thrombosed.

## Discussion

This study explored the clinical course, radiological, laboratory, and periprocedural parameters, and the functional outcome of patients with VITT-associated CVST with space-occupying effects and DS in direct comparison with patients suffering from non-VITT CVST also requiring DS. The main finding of this study was that patients with VITT-associated CVST had a similar clinical course before DS compared with patients with non-VITT CVST. On the other hand, patients suffering from VITT-associated CVST more frequently achieved a favorable functional outcome after 3 months. Remarkably, despite the immune-mediated activation of the coagulation system, substitutions (e.g., platelets) in patients with VITT did not result in an increased rate of complications compared with patients with non-VITT CVST.

Until 2021, CVST not related to VITT was mostly seen as a relatively benign disease, as most patients in large registries exhibited only mild neurological deficits (e.g., baseline NIHSS score of less than 5 in 90% of patients, Glasgow Coma Scale score of less than 9 in only 0.9–5.2% of patients) [4, 5]. The necessity for DS as an ultima ratio option in severe CVST with space-occupying effects due to edema or bleeding complications was low and ranged from 0.9 to 1.4% of patients [4, 5]. However, because of the low number of patients with severe CVST, knowledge on effects related with DS was limited to retrospective and mostly single-center studies rather than multicenter approaches [6, 8, 24]. In contrast, an earlier report indicated that patients with VITT-associated CVST differed dramatically from patients with non-VITT CVST: In detail, a severely decreased level of consciousness (coma in 24% of patients) and intracerebral bleedings (in 68% of patients) were frequently observed at hospital admission, and mortality during the hospital course was high [6]. Accordingly, in a single-center, retrospective study, the decision for surgery in CVST was taken at admission or within the first 12 h from hospital admission (47%) in severe VITT-associated CVST [14].

Furthermore, this study demonstrated that clinical symptoms and other characteristics in patients with VITT-associated and non-VITT CVST, both undergoing DS, were comparable at hospital admission, as demonstrated by similar NIHSS, level of consciousness, APACHE 2, SAPS 2, and radiological findings. However, both groups significantly deteriorated between admission and surgery, which is likely due to a progressive space-occupying effect of CVST, although a relevant midline shift of ≥ 4 mm before surgery was found more frequent in patients with VITT-associated CVST. This might be the reason why a higher proportion of patients with VITT-associated CVST underwent early DS, i.e., within 24 h after hospital admission, compared with non-VITT CVST. However, a rapid neurological deterioration was also reported in a former study on patients with non-VITT CVST, with a mean interval of 24 h from diagnosis of CVST to the first appearance of signs for a space-occupying admission in 54.5% of patients, primarily because of a midline shift of ≥ 10 mm [25]. In general, CVST with space-occupying effects may be described as a vicious circle: The intracerebral mass effect of the (congestive) hemorrhage and edema, respectively, leads to an increase of the ICP, which further reduces the venous outflow, leading to venous stasis, and thus facilitating the propagation of thrombosis. In patients suffering from VITT, this might be further aggravated by the concurrent massive activation of the coagulation system. Although anticoagulation and immunomodulatory treatment in patients with VITT-associated CVST was initiated timely, platelet count and D-dimer before surgery were similar to admission. At the same time, fibrinogen even decreased, which is consistent with an ongoing activation of the coagulation system.

Despite the massive activation of the coagulation system resulting in restricted hemostasis, this study did not observe a higher frequency of perioperative complications in patients with VITT-associated CVST. This was most likely related to the perioperative administration of thrombocyte concentrates, tranexamic acid, and fibrinogen to optimize hemostasis before and during surgery. Generally, it is recommended to be cautious with substituting platelets in autoimmune-mediated thrombocytopenias like heparin-induced thrombocytopenia type 2 as they might precipitate thrombotic events [26, 27]. However, evidence is weak as retrospective case series reported no increased risk for thrombotic events after platelet transfusion [28, 29]. Hence, from the neurosurgical point of view, the risk of excessive bleeding during surgery in the case of low platelets and also swollen sinus and cortical veins because of the severely impaired venous outflow probably outweighed the possible prothrombotic risk of a platelet transfusion. Accordingly, no differences were found for the transfusion of erythrocyte concentrates or volume replacement during surgery, which, in the proper sense, served as indicators for an increased intraoperative loss of blood. In both groups, intracranial rebleeding after surgery was frequent and occurred in more than every second patient, with a comparable necessity for further neurosurgical interventions. Data on complications after DS are rare, and the reported frequency considerably varies [30]. The few available data indicate that significant complications may occur immediately in the postoperative course in about 20% of patients with CVST and DS [25]. Ban and colleagues reported complications secondary to decompressive craniectomy in traumatic brain injury in 48 of 89 (53.9%) patients [31], while Kurland and coworkers [32] concluded in their review that about 10% of all patients undergoing decompressive craniectomy might suffer a complication necessitating additional medical and neurosurgical intervention, respectively.

Concerning the course in the neuro-ICU, patients with VITT-associated and non-VITT CVST were treated in a comparable way. In detail, most patients received three or more drugs for analgesia and sedation simultaneously, probably to control an ongoing increased ICP. Duration of analgosedation and duration of artificial ventilation tended to be shorter in patients with VITT-associated CVST, which might indicate a better short-term outcome compared with patients with non-VTT CVST. Key actions of the applied intensive care were also comparable between both groups. Almost all patients had invasive ICP monitoring, and more than half had external ventricular drainage and received osmotherapy. Although in patients with space-occupying hemispheric infarction DS is clearly indicated, and osmotherapy is often reserved for cases in which surgery is insufficient to control the ICP [7], it seems to be vice versa in CVST: In the French Prospective Cohort of patients with Cerebral Venous Thrombosis (FPCCVT) only 0.9% of patients underwent DS, while 19.9% received osmotherapy [5]. In a multicenter, retrospective German study including only patients with severe (Glasgow coma scale ≤ 9) CVST, 40.4% of patients received osmotherapy, and only 19.3% had DS [33]. The rate of osmotherapy was even higher in both groups of this study, with up to 75% in patients with VITT-associated CVST, which might be attributed to an insufficiently controlled ICP despite surgery.

In the present study, only a few patients had targeted temperature management, which is likely related to the fact that earlier studies did not show beneficial effects in patients with space-occupying infarction [34] and intracerebral hemorrhage [35]. Both groups had comparable complications during treatment in the neuro-ICU like severe sepsis, ventilator-associated pneumonia, liver failure, and acute kidney injury. Further, the frequency of extracranial thrombosis did not differ between both groups.

Our results must be interpreted with caution, even though patients with VITT-associated CVST were seen with a significantly better functional outcome after 3 months compared with patients without VITT. Large registries including patients with non-VITT CVST indicated high rates of an excellent functional outcome (mRS 0 or 1) after a median of 12 and 16 months: 88.3% in the FPCCVT cohort and 87.4% in the International Study on Cerebral Vein and Dural Sinus Thrombosis (ISCVT) cohort [4, 5]. While focusing on patients with CVST and subsequent DS, rates of favorable functional outcome (mRS ≤ 3) were comparable between single-center, retrospective studies with 65.9% after 6 months [25] and 77.6% after 6 months [9], and a registry with 73.7% after a median of 14.5 months [24]. However, in patients with severe CVST, as indicated by a Glasgow Coma Scale ≤ 9 at hospital admission or during the hospital stay, a favorable outcome (mRS ≤ 3) after 3 to 9 months was achieved by 39.9% of patients [33], which is in good accordance with patients suffering from VITT-associated CVST in the present study. On the other hand, mortality was high (62.5%) in this study among patients with non-VITT CVST and DS, contrasting with mainly retrospective, single-center studies that reported mortality rates of no more than 30% [8, 37]. Considering again former data from Germany, Kowoll et al. [33] reported a mortality of 34% in patients with severe CVST (with and without DS). Because of the rarity of CVST with space-occupying effects before the occurrence of VITT-associated CVST, the decision for DS is typically made on an individual basis. Notably, the optimal time point of surgery is unclear, as the guidelines only state to operate in case of “impending herniation to prevent death” [3]. So far, DS in patients with severe CVST has been considered as an ultima ratio approach. This attitude might have changed in 2021, when many patients were affected by severe CVST in a short time period. Consequently, patients with VITT-associated CVST underwent surgical treatment faster after admission than patients with non-VITT CVST. Remarkably, the situation is entirely different for patients with space-occupying brain infarction, where a hemispheric infarction involving about two thirds of the vascular territory of the middle cerebral artery and a disturbance of consciousness (at least somnolence) represents a clear indication for DS, which means that the surgical procedure should be done before any signs of herniation occur [36]. This might also help to explain the discrepancy between the high rate of osmotherapy and the low rate of DS in patients with CVST [4, 5]. Because of the poorly defined optimal time point for surgery, clinicians may still consider DS as an ultima ratio approach in patients with CVST, reserved for the situation when the conservative treatment comprising analgosedation and osmotherapy has failed, and the risk for death due to herniation is high. However, as considerably high rates of a favorable outcome can also be achieved in severe CVST and since the prediction of the individual course is challenging with a relevant risk of sudden deterioration, DS should be considered early, particularly in patients with VITT.

This study has some limitations: Firstly, due to the rarity of VITT-associated CVST with space-occupying effects and consecutive DS, this study is based on a relatively small number of patients. However, a multicenter approach was used to address this issue, which has led to a total of 32 patients, including the non-VITT group, which is comparable with former studies on CVST [8]. Applying a period of 15 years (1996 to 2011), Kowoll and colleagues [33] included at least 114 patients from ten tertiary centers with severe CVST, of whom only 23 underwent DS. Additionally, in large multicenter cohorts the rate of DS in CVST was low, ranging between 0.9% (FPCCVT in France [5]) and 1.4% (ISCVT [4]). Considering an annual incidence in Germany of 1.9 CVST per 100,000 people [2], one would expect a total of 1,577 CVST cases per year in Germany. Over the study period of 9.5 years, this would result in about 15,000 CVST cases. Assuming a rate of DS of 0.9% like in France [5], one would expect 135 cases of non-VITT CVST undergoing DS in Germany between 2012 and 2021, of whom 16, that is, 11.9% or every 8th patient, was included in this study. Nevertheless, most results of the present study do not even show a trend for one of both groups, making it plausible to assume that this study would not lead to other findings in case of more patients. Secondly, assessing the functional outcome after 3 months might be too early. Although the time point of three months is frequently used in stroke studies (e.g., in most studies examining mechanical thrombectomy), an earlier report indicated that patients with severe CVST and DS demonstrate further functional improvement beyond 3 months, i.e., after 6 and 12 months [25]. However, the timing of endpoints in retrospective studies is difficult. It often results in a time range rather than a specific time point, e.g., 3 to 9 months in the study by Kowoll and colleagues [33]. Thirdly, because of the limited sample size, we could not investigate the impact of clinical factors (such as midline shift, fixed pupils, or coma before surgery), neither of radiological factors of the preoperative or postoperative cerebral imaging. In particular, new hemorrhagic lesions on postoperative imaging may be associated with poor clinical outcome in patients with non-VITT [38]. Finally, this study used a retrospective design. However, we postulated the hypotheses a priori and designed the case report form to address these hypotheses. In addition, the data collection was relatively complete because most parameters were reported in > 80% of patients. Because of the rarity of VITT-associated CVST and the actions that have been done to reduce its occurrence, e.g., the restriction of related vaccines to age cohorts, a prospective trial that compares, for example, the optimal timing for DS or against conservative treatment would not be feasible. Noteworthy, vector-based vaccines against SARS-CoV 2 are still in use outside Europe and the USA. Recently, fatal courses of VITT-associated CVST were reported after vaccination with the Sputnik V vaccine [39], and VITT may also occur after vaccination with the Sinopharm vaccine [40]. Thus, the findings of this multicenter cohort study may help clinicians in treating patients with VITT-associated CVST, independent of the vaccinated vector type.

In conclusion, this study provides a multiparametric characterization of patients suffering from severe VITT-associated CVST with a need for DS in direct comparison with non-VITT CVST also requiring DS. Thereby, patients suffering from VITT-associated CVST more frequently achieved a favorable functional outcome after three months. DS should thus be considered early in VITT-associated CVST.
