# Correction: Outcomes After Decompressive Surgery for Severe Cerebral Venous Sinus Thrombosis Associated or Not Associated with Vaccine-Induced Immune Thrombosis with Thrombocytopenia: A Multicenter Cohort Study

**DOI:** 10.1007/s12028-025-02354-6

**Published:** 2025-08-18

**Authors:** Johann Otto Pelz, Martin Kenda, Angelika Alonso, Nima Etminan, Matthias Wittstock, Wolf-Dirk Niesen, Johann Lambeck, Erdem Güresir, Johannes Wach, Tim Lampmann, Rainer Dziewas, Markus Wiedmann, Hauke Schneider, Antonios Bayas, Monika Christ, Annerose Mengel, Sven Poli, Dirk Brämer, Dirk Lindner, Christian Pfrepper, Christian Roth, Farid Salih, Albrecht Günther, Dominik Michalski, for the IGNITE Study Group

**Affiliations:** 1 Department of Neurology, University Hospital Leipzig, Liebigstrasse 20, 04103 Leipzig, Germany; 2 Department of Neurology and Experimental Neurology, Charité-Universitätsmedizin Berlin, Campus, Virchow-Klinikum, Berlin, Germany; 3 Department of Neurology, Medical Faculty Mannheim, University of Heidelberg, Mannheim, Germany; 4 Department of Neurosurgery, Medical Faculty Mannheim, University of Heidelberg, Mannheim, Germany; 5 Department of Neurology, University Medicine Rostock, Rostock, Germany; 6 Department of Neurology and Clinical Neurophysiology, University Medical Center Freiburg, University of Freiburg, Freiburg, Germany; 7 Department of Neurosurgery, University Hospital Bonn, Bonn, Germany; 8 Department of Neurosurgery, University Hospital Leipzig, Leipzig, Germany; 9 Department of Neurology and Neurorehabilitation, Klinikum Osnabrueck, Osnabrueck, Germany; 10 Department of Neurosurgery, Oslo University Hospital, Oslo, Norway; 11 Department of Neurology, University Hospital Augsburg, Augsburg, Germany; 12 Department of Neurology and Stroke, University Hospital Tuebingen, Eberhard-Karls University, Tuebingen, Germany; 13 Department of Neurology, Jena University Hospital, Jena, Germany; 14 Division of Haemostaseology, Medical Department I, University Hospital Leipzig, Leipzig, Germany; 15 Department of Neurology, Klinikum Kassel, Kassel, Germany; 16 Medizinische Fakultät Carl Gustav Carus, Technische Universität Dresden, Dresden, Germany

**Correction to: Neurocritical Care (2023) 40:621–632** 10.1007/s12028-023-01782-6

The original article has been corrected. Dr. Lampmann's surname has been updated to Lampmann instead of Lampmannn.

